# ﻿A new species of *Hoya* R.Br. (Apocynaceae, Asclepiadoideae) from the Philippines

**DOI:** 10.3897/phytokeys.222.98275

**Published:** 2023-03-20

**Authors:** Miguel David De Leon, Derek Cabactulan, Ryu D. Cuerdo, Michele Rodda

**Affiliations:** 1 Cagayan de Oro Medical Center, Tiano cor. Nacalaban Sts., Cagayan de Oro, Misamis Oriental 9000, Philippines Cagayan de Oro Medical Center Cagayan de Oro Philippines; 2 48 Corrales and 1st Streets, Nazareth, Cagayan de Oro City, Misamis Oriental 9000, Philippines Unaffiliated Cagayan de Oro Philippines; 3 Tejero, San Luis, Batangas 4210, Philippines Unaffiliated San Luis Philippines; 4 Singapore Botanic Gardens, National Parks Board, 1 Cluny Road, Singapore 259569, Singapore Singapore Botanic Gardens Singapore Singapore

**Keywords:** *Hoyaedeni*, *Hoyalinavergarae*, *Hoyaodorata*, Luzon, Marsdenieae

## Abstract

*Hoyamedusa* M.D.De Leon, Cabactulan, Cuerdo & Rodda, **sp. nov.** (Apocynaceae, Asclepiadoideae) is described from the Philippines. Even though numerous taxa with a shrubby habit from this area are known, it can be immediately separated because of its urceolate corolla and prominent elongated corona lobes. No other species in the genus possesses such a combination of characters.

## ﻿Introduction

*Hoya* R.Br. is a megadiverse genus occurring from the Himalayan foothills to Okinawa (Japan) and through Southeast Asia and Malesia to Australia and the Fiji Islands. Despite being fairly well known in Thailand (Thaithong et al. 2018), Vietnam (Tran 2017), Singapore (Middleton and Rodda 2019), Borneo (Lamb and Rodda 2016; Rodda and Rahayu 2022) and New Guinea (Cámara-Leret et al. 2020; Rodda and Simonsson 2022), much is still needed to complete larger revisions and incorporate in keys the wealth of new species which keep being described at a fast pace, making published revisions quickly outdated.

Based on the available data (Pelser et al. 2011 onwards; Lamb and Rodda 2016; Cámara-Leret et al. 2020; Rodda and Rahayu 2022; Rodda and Simonsson 2022) the centres of diversity of *Hoya* are Borneo, New Guinea and the Philippines. Species numbers for Borneo and New Guinea will likely keep increasing as new species are described (Rodda and Rahayu 2022; Rodda and Simonsson 2022) but since most species are well known we do not foresee large numbers of synonyms. In the Philippines, Pelser et al. (2011 onwards) list as many as 202 *Hoya* taxa, but state that ‘The number of *Hoya* species in the Philippines is doubtlessly much lower than suggested by this account’. We agree that many names will become synonyms once the genus is revised. Based on IPNI [https://www.ipni.org, accessed on 2 Dec 2022], since 2010, 352 new *Hoya* taxa were published, of which 165 from the Philippines. However, most of these names have been published in non-peer-reviewed publications and their types shall be carefully examined before they can be considered accepted taxa. For most of these names, descriptions are brief, the illustrations provided with the publication are insufficient, and type citation is generally limited to a herbarium sheet number which upon closer examination might be a collector number or even the collection date of the specimen (Rodda 2015; Rodda 2017).

Within the past 20 years numerous species with a shrubby or pendulous (non-twining) habit have been published in *Hoya* (Rahayu and Rodda 2020), and many of them come from the Philippines: *Hoyaamrita* Kloppenb., Siar & Ferreras, *H.cembra* Kloppenb., *H.cumingiana* Decne., *H.densifolia* Turcz., H.densifoliaTurcz.subsp.panchoi Kloppenb., *H.golamcoana* Kloppenb., *H.irisiae* Ferreras, Kloppenb. & Tandang, *H.lazaroi* Kloppenb. & Siar, *H.linapauliana* Kloppenb., *H.linavergarae* Kloppenb. & Siar, *H.maquilingensis* Kloppenb., *H.multiflora* Blume, *H.nova* Kloppenb., *H.odorata* Schltr. (with six recently published subspecies), *H.paziae* Kloppenb., and *H.platycaulis* Simonsson & Rodda.

Many of these names will require extensive investigations to verify if they are applicable.

Yet, extraordinary novelties can still be found in the Philippines. In 2018, plants of a shrubby species were found in cultivation. Based on photographs of the flowers, the last author initially thought that this species might be a member of Apocynaceae subfamily Periplocoideae due to the elongated corona lobes (which were mistaken for anther appendages). Upon closer examination of the corona and pollinarium it became clear that it nevertheless belongs to *Hoya* as the corona lobes have basal revolute margins, and the pollinia have the distinct sterile edges diagnostic of *Hoya* (Wanntorp and Kunze 2009). The urceolate corolla combined with prominent elongated corona lobes make this taxon unique among all species of *Hoya*. Comparison with previously described species of *Hoya* was carried out in person or via loans or photographs from A, BM, BO, FI, K, LAE, MO, P, PNH, SING, and UC herbaria (Thiers 2023).

## ﻿Species treatment

### ﻿ Hoya medusa

Taxon classificationPlantaeGentianalesApocynaceae

M.D.De Leon, Cabactulan, Cuerdo & Rodda sp. nov.

77B9CB2B-847B-5F4F-B42B-C8E18EA42DF7

urn:lsid:ipni.org:names:77315879-1

Figs 1 , 2

#### Diagnosis.

Similar to *Hoyaedeni* King & Hook.f. in its shrubby habit and caudate and curved inner corona lobe processes, distinct by the corona processes (wavy to serpentine in *H.medusaversus* linear and curved at the tip in *H.edeni*), corolla shape (urceolate in *H.medusaversus* rotate with reflexed lobes in *H.edeni*) and size (8.5–10 mm in *H.medusaversus* c. 20 mm in *H.edeni*).

#### Type.

Philippines. Luzon, Cagayan Province, Mt. Cetaceo 500 to 1,000 m, vouchered in cultivation, 07 Feb 2022, *M.D. De Leon s.n.* (holotype: PNH, sheet no. 258696).

#### Description.

***Plant*** epiphytic pendent shrub, with white exudate in all vegetative parts. ***Roots*** basal, fibrous. ***Stems*** slender, terete, 1.5–7 mm in diam., green, sparsely puberulent when immature, turning lignified, brown and glabrescent when mature, ***internodes*** 2.8–6 cm long. ***Leaves***: petiole stout, usually curved, canaliculate above, flattened towards lamina base, 3–7 mm long, 1.5–2.0 mm in diameter, dark green, sparsely pubescent turning glabrescent when old; blades coriaceous, stiff, flat to slightly curved, variable in shape, oblong, ovate, to elliptic-ovate, 55–90 mm long × 20–34 mm wide; base acute to obtuse, apex acute to acuminate, with a caudate tip, adaxially mid-green, abaxially light green; margins entire, occasionally slightly undulate; adaxially and abaxially sparsely pubescent to glabrescent in older leaves; venation pinnate-brochidodromous, with 4–8 lateral veins on each sides of mid vein, prominent (very pale green) on younger leaves and obscure on older leaves yet clearly visible on dried specimens; colleters one at each lamina base, conical, c. 0.20 mm long, grayish brown. ***Inflorescence*** extra-axillary, umbelliform, convex, with up to 11 flowers (occasionally up to 16 flowers in particularly strong cultivated specimens). ***Peduncle*** short stout or sometimes sessile, cylindrical, 1.0–3.0 mm long and 1.0–2.0 mm in diam., perennial, sparsely pubescent. ***Pedicels*** terete, outer pedicels slightly curved, otherwise straight, 8–10 mm long and 0.5–0.8 mm in diam., light green, lenticellate except the base of the calyx which is pubescent. ***Calyx*** lobes oblong, 1.8–2 mm long, c. 0.50 mm wide, reddish, abaxially strigose, adaxially glabrous, basal colleters 1 between each calyx lobes, oblong, c. 0.02 mm long × c. 0.02 mm wide. ***Corolla*** basally urceolate, with flat spreading lobes, white, corolla lobes spreading, 8.5 to 10 mm in diam. across the corolla lobes, corolla tube 2.0–3.0 mm long, 4–5 mm in diameter, lobes triangular ovate, 2.5–3.0 mm long, 2.5–3.5 mm wide, apex acute, slightly revolute, margins revolute, corolla lobes adaxially densely strigose, densely hirsute towards the rim and interior of the tube and the column, abaxially glabrous; ***gynostegium*** stipitate; ***column*** cylindrical, 0.50–0.70 mm high × c. 0.50 mm in diam.; ***corona*** staminal 4–5 mm high, c. 3 mm in diam.; ***corona lobe*** bulbous-obpyriform, c. 3.5 mm long × 0.5–0.7 mm wide, inner (apical) process caudate, upright, curved, wavy to serpentine, meeting at the center and overlapping, erect above the gynostegium, outer (basal) process obovate, with basal revolute margins, guide rail raised, laterally compressed, prominent at the base of the corona lobes c. 0.80 mm long and extending c. 0.20 mm laterally. ***Pollinia*** erect, elliptic-oblong, c. 0.50 mm long, c. 0.22 mm wide; ***caudicle*** obovoid, c. 0.10 mm diam; ***corpusculum*** oblong, c. 3.0 mm long by c. 1.0 mm wide; ***ovary*** ovoid, c. 1.5 mm long, each carpel c. 0.9 mm wise at the base, glabrous. ***Fruit and seed*** not seen. Flowers vespertine lasting up to 15 days in cultivation, drying of the inner corona processes begins on the second day causing brown to black discoloration, flowers slightly fragrant, fragrance floral, powdery scent or mild jasmine persistent throughout the day and night.

**Figure 1.**
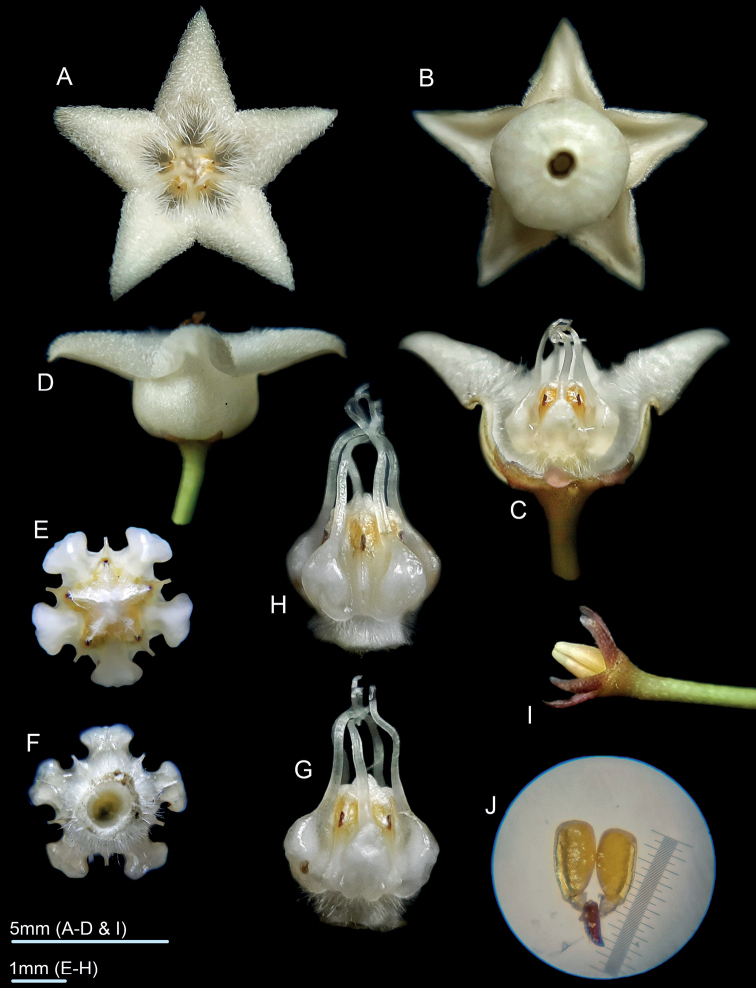
*Hoyamedusa* photographed in cultivation **A** flower, top view **B** corolla, from underneath **C** flower, side view two corolla lobes removed **D** flower, side view **E** corona, top view **F** corona, from underneath **G, H** corona, side view **I** calyx and ovaries **J** pollinarium. (Photographs by R.D. Cuerdo).

#### Etymology.

The specific epithet refers to the serpentine processes of the inner corona reminiscent of the snake headdress of Medusa in Greek mythology.

#### Distribution and ecology.

*Hoyamedusa* was collected by local collectors in Luzon Island, Mt. Cetaceo and has been in cultivation, circulated by local plant nurseries and plant hobbyists. It was first collected in low montane forest at 500 to 1,000 m where it was growing as an epiphyte in disturbed primary broadly leaf and mossy forest in full sun to part shade.

**Figure 2.**
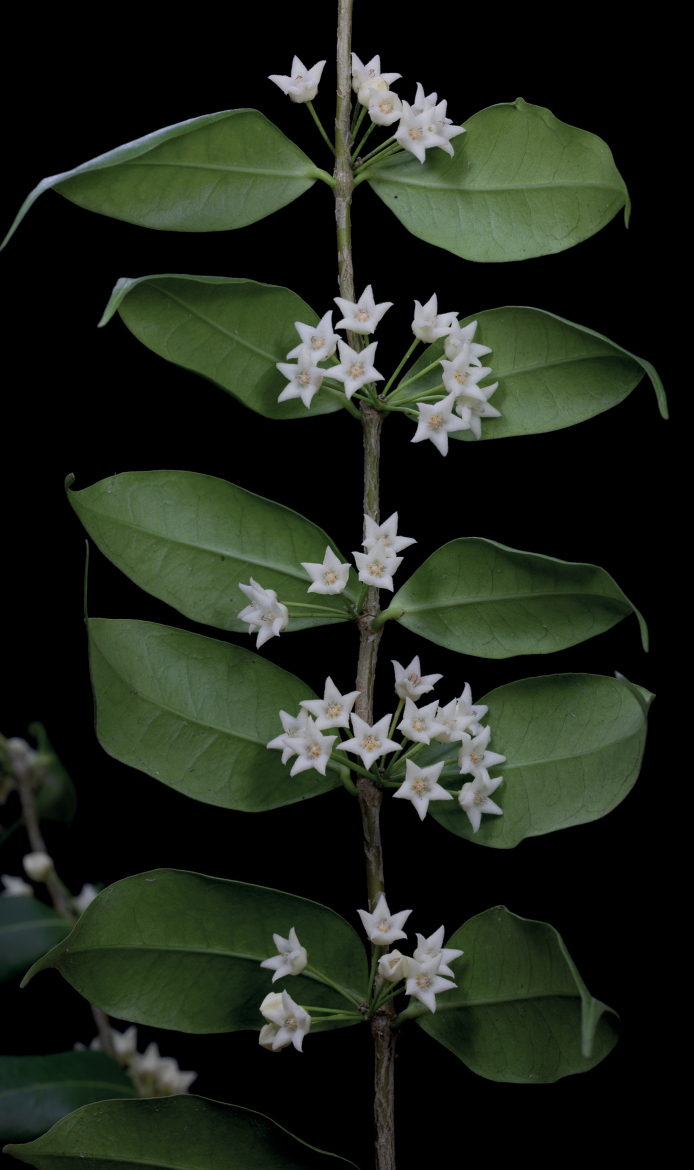
*Hoyamedusa* flowering branch. (Photograph by M.D. De Leon).

#### Conservation status.

The forested area where *Hoyamedusa* was collected is threatened by habitat destruction due to extensive harvesting of trees for local housing and wood-fuel use, destructive farming practices such as “*kaingin*” (slash and burn farming in Filipino), land conversion and illegal commercial logging. The type locality is a heavily logged forest. Parts of the deforested areas had been converted to agricultural land for crops such as corn, cassava, and vegetables. The western portion of the area is near a protected forested area and remains intact but it is endangered by rapid expansion of agricultural lands by the lowland settlements.

*Hoyamedusa* is only known from a single collection and we do not have information on the extant population size; therefore, the conservation status is proposed as Data Deficient (DD, IUCN Standards and Petitions Committee (2022)) until more information is known about its area of occurrence.

#### Notes.

*Hoyamedusa* displays an unusual combination of characters, in particular shrubby habit and corolla urceolate, with flat spreading lobes. This sets it apart from all other shrubby species of *Hoya*. In the diagnosis we compared it to *Hoyaedeni* because both species are shrubs and present somewhat similar inner corona lobe processes, yet they present very different corolla morphology. The only other species with a similar corolla shape (salverform, with tube narrowly campanulate) is *Hoyakachinensis* Rodda & K.Armstr. from Myanmar, which is a climber with oblanceolate, ca. 25 long leaves and therefore clearly distinct from *H.medusa*. Other species that have a somewhat similar corolla shape are *Hoyatelosmoides* Omlor from Borneo, and *H.versteegii* Simonsson & Rodda from New Guinea, which are both twining climbers. The only species with a tubular/urceolate corolla, somewhat spreading but much reduced corolla lobes as well as a shrubby habit is *Hoyamanipurensis* Deb from India, Myanmar, China and Thailand. *Hoyamanipurensis* has obcordate to triangular laminas and has corolla lobes much shorter than corolla tube, making it once again very easily separated from *H.medusa*.

Among other species of *Hoya* occurring in the Philippines *Hoyamedusa* is somewhat similar to *Hoyalinavergarae* and *Hoyaodorata*. The leaf margins of *H.medusa* are slightly undulate, whereas undulate in *H.linavergarae*, and flat in *H.odorata*. The flowers of *H.medusa* are smaller (8.5 to 10 mm) and basally urceolate with spreading lobes, whereas *H.linavergarae* and *H.odorata* have larger flowers (15–23 mm and 13–18 mm respectively), and basally campanulate (*H.linavergarae*), or rotate (*H.odorata*) with inflexed corolla lobes. Further, the corolla of *H.medusa* is densely hirsute inside, whereas puberulent in *H.linavergarae* and *H.odorata*. The inner corona processes of *H.medusa* have a caudate, upright, curved, wavy to serpentine appendage, whereas the inner corona processes of *H.linavergarae* and *H.odorata* do not have an appendage.

## Supplementary Material

XML Treatment for Hoya medusa
